# Site-selective cleavage of peptides and proteins targeting aromatic amino acid residues

**DOI:** 10.1039/d4ra08956a

**Published:** 2025-03-25

**Authors:** Ayan Bandyopadhyay, Rajib Sarkar

**Affiliations:** a Department of Chemistry, Chapra Government College Nadia-741123 West Bengal India; b Department of Higher Education, Government of West Bengal India rajibsarkar.org@gmail.com; c Department of Chemistry, Muragachha Government College Nadia-741154 West Bengal India

## Abstract

The site-selective cleavage of peptides and proteins at specific amino acid residues is an important strategy for the modification of biomolecules as it can potentially transmute the reactivity profile of the whole molecule. Moreover, precise cleavage of a specific amide bond in peptides and proteins has enormous applications in the domains of chemical biology, genetics, and protein engineering. Among the 20 proteinogenic amino acids, tryptophan (Trp, W), tyrosine (Tyr, Y), phenylalanine (Phe, F) and histidine (His, H) are classified as aromatic amino acids that maintain the function of protein folding through hydrophobic and π–π interactions. Thus, scissoring at a specific site of an aromatic amino acid may alter the structure and function of a peptide or protein. In the last 60–70 years, great success has been achieved in the development of methods for the aromatic amino acid (AAA)-selective cleavage of peptides and proteins. Generally, aromatic side chains are derivatized in the presence of specific reagents. Consequently, either the downstream or the upstream amide bond of the aromatic side chain is activated, and hydrolysis of the amide bond splits the peptide. Unfortunately, a systematic review covering this methodological development of the AAA-selective fission of peptide is lacking to date. Thus, in this review, we aim to showcase the up-to-date progress in the site-selective rupture of peptide bonds at aromatic amino acid residues with an emphasis on the postulated mechanisms, enabling future researchers to further drive progress in this research field.

## Abstract 2

This review provides an account of the site-selective cleavage of peptides and proteins at the aromatic amino acid residues, developed over the last seven decades (1958–2024).

## Graphical abstract

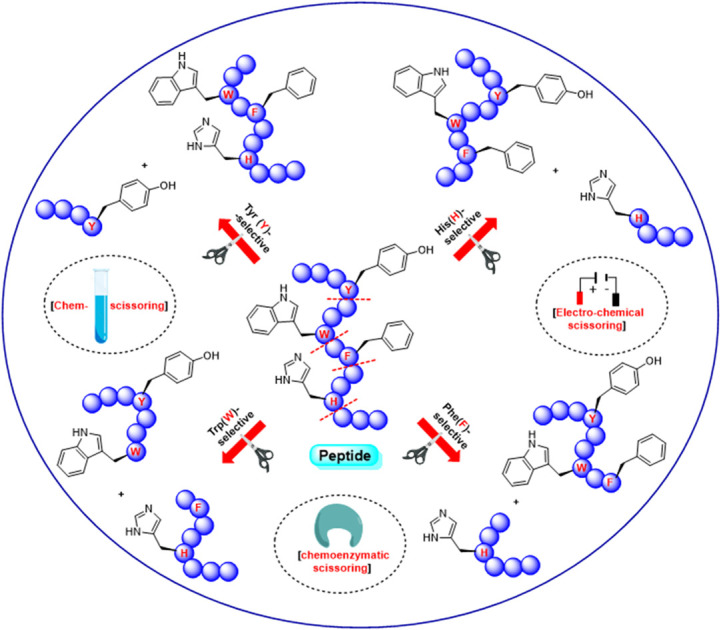

## 1. Introduction

Modification of a polypeptide in a specific amino acid residue can unlock a variety of chemical compounds with structural diversities and profound biological activities. Alteration of peptides and proteins can be achieved by installing new functional groups or through the bio-conjugation of small molecules into the core of amino acid residues and proteins,^1–7^ cyclization of polypeptides^8–10^ or cleaving long peptides or proteins.^11–14^ Among these modification techniques, the site-specific cleavage of peptide linkages is a noteworthy tool, which has exhibited promising applications in proteomics,^15^ site-selective derivatization,^16,17^ and design of novel therapeutics.^18–22^ Fission of peptide bonds produces new peptide fragments with a modified N- or C-terminus. Sometimes, some of the side chains of the residues are also modified during the cleaving process. Generally, these alterations metamorphose a molecule into a reactive intermediate, which can be further functionalized as drug leads and therapeutic agents.^17,23^ Moreover, the cleavage of the peptide bonds at specific residues followed by amino acid analysis or liquid chromatography with tandem mass spectrometry (LC-MS/MS) is a classical technique for the determination of the sequence of unknown peptides and proteins.^24^

Amide bonds are one of the most fundamental and crucial chemical bonds in nature for the existence of life. They are very stable, exhibiting a half-life of approximately 350–600 per year for spontaneous hydrolysis at room temperature (RT) and neutral pH.^25^ As a result, conventional protocols for amide hydrolysis require harsh reaction conditions.^26^ However, nature can cleave peptides and proteins through various enzymatic processes under physiological conditions.

Aromatic amino acid (AAA) residues are some of the most important segments of proteins. Biomolecules that contain AAA, possess some unique properties and functions. Thus, the rupture of selective sites in the AAAs of peptides and proteins has long been a topic of interest for chemists and biologists. This particular research area emerged in the 1950s. The aromatic side chains of W-, Y-, F- and H-residues were targeted with chemoselective reagents, activating their derivatives at either the upstream or downstream amide bond. For example, oxidative halogenation of the side-chains of the W- and Y-residues can unlock the activation of the downstream amide bond. Alternatively, co-ordination of the imidazole side-chain of the H-residue with suitable metal ions can activate both the upstream and downstream amide bond depending upon the reaction conditions. Thus, the residue-specific and chemoselective scissoring of amide bonds became possible. Thereafter, numerous non-enzymatic protocols were developed gradually. Unfortunately, this research field was neglected for a long time. However, with the discovery of new proteins and the utilization of fragmented proteins as drug leads, recently this field has emerged again. Lots of protocols for the site-selective scissoring of peptides have been developed in the last two decades. An elegant book chapter was contributed by the Kanai group, which summarized the chemical approaches utilized in the site-selective cleavage of peptides and proteins over the last 10 years (2005–2015).^27^ Although not specifically focused on all AAAs, in 2023, the Brimble group published a review, in which a small section addressed the latest advances in the selective cleavage of peptides and proteins at the tyrosine sites only.^28^ The advancements in the site-selective cleavage of peptides at the AAA residues in recent years as well as their enormous application in the fields of drug design and proteomics necessitate an up-to-date summary of the current state of this field. We hope that this article will quench the thirst of young researchers.

## 2. Importance of site-selective cleavage of peptides at aromatic amino acid residues

**Fig. 1:**
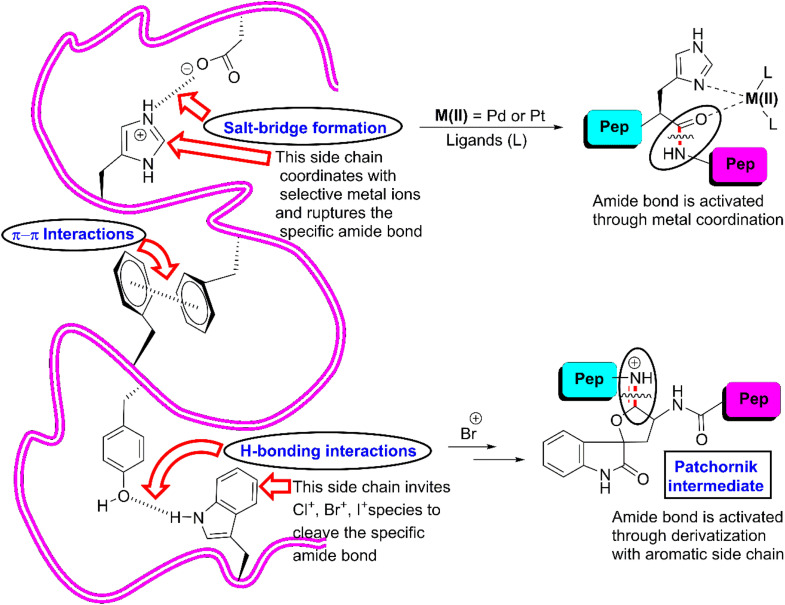
Key interactions exerted by AAAs that fix a polypeptide chain as well as activate amide bonds.

As is known, among the 20 proteinogenic amino acids, the W-, Y-, F- and H-residues are categorized as AAA given that they each contain a side chain that is connected with an aromatic moiety. These residues are often acquired in helical and β-barrel membrane proteins and possess unique physicochemical properties. Their presence is very important for protein–protein interactions (PPI) in biological systems.^29,30^ The indolyl side chain in the W-residue contributes to PPI through π-interactions, hydrophobic interactions and hydrogen bonding.^31^ Thus, it has the potential to bind with a large number of residues simultaneously owing to its comparatively larger size than the other residues.^32^ Alternatively, the phenolic side chain of the Y-residue is amphiphilic in nature. Its hydroxyl group enhances the hydrophilic character by constructing hydrogen bonds, while its aromatic counterpart creates a balance by contributing hydrophobic interactions. In the context of PPI, the presence of the F-residue is indeed interesting. Despite its phenyl ring, its frequency in PPI is still one-third that of the Y-residue. The imidazole-tie knotted H-residue can interact with nonpolar and aromatic groups with its heteroaromatic moiety, while its heteroatoms can be involved in hydrogen bonding. Utilizing various protonated states of the imidazole segment, it can be engaged in salt-bridges with acidic groups of other biomolecules^33^ (Fig. 1). Actually, AAAs force the polypeptide chain to adopt a specific 3-dimensional structure through various covalent interactions such as hydrogen bonding, π–π stacking interactions, and hydrophobic interactions. Besides, they can induce interactions with specific reagents to. For example, W- and Y-residues react with *N*-bromosuccinimide (NBS) to afford the corresponding bromo-derivative that activates the downstream amide bond to cleave (Fig. 1). Similarly, H-residues can co-ordinate with selective metal ions to break selective amide bonds. Undoubtedly, AAAs play crucial roles and dictate the function of proteins in biological systems. Thus, scissoring at that specific site can snatch numerous inner information from the core of protein molecules.

## 3. Advances in site-selective cleavage of peptides at aromatic amino acid residue

### 3.1. Tryptophan

Tryptophan (Trp, W) is an essential amino acid, which is inserted into protein molecules by encoding the codon UGG. The W-residue is rare in proteins, but it plays significant structural or functional roles whenever it appears.^34^ Thus, the investigation of the specific function of W-residues in peptides and proteins is of growing interest to chemists as well as biologists. In this context, W-selective cleavage by means of various chemical, electrochemical, or chemoenzymatic techniques, followed by deep analysis may solve these purposes. The strategies that promote the W-selective fission of peptide bonds in peptides are showcased below.

#### 3.1.1. Chemical method

**Fig. 2:**
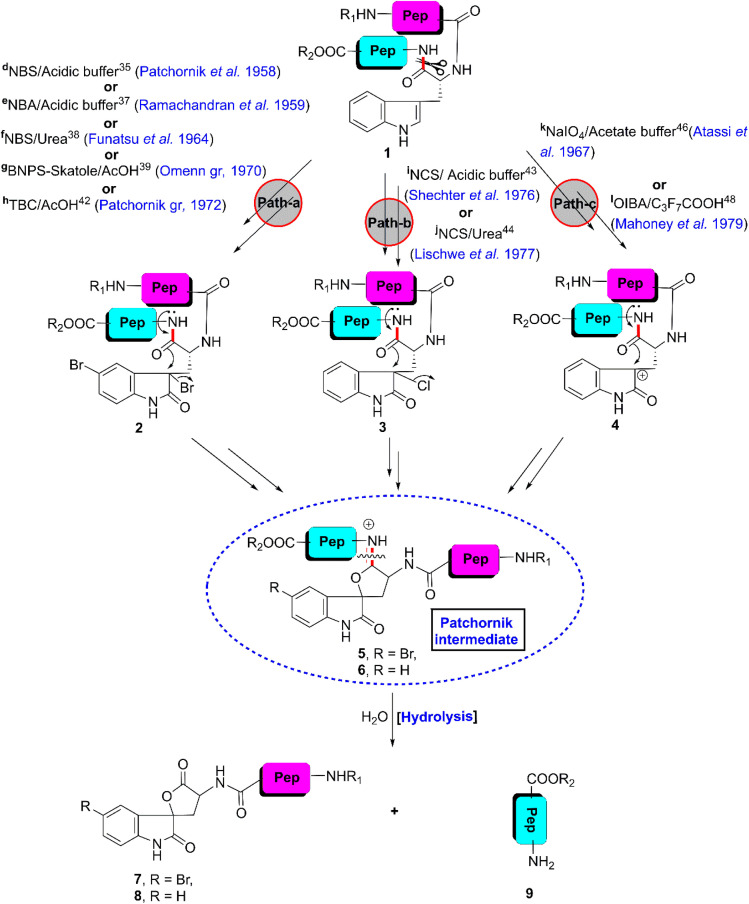
W-selective scissoring of peptides/proteins through chemical oxidation. [Reaction conditions: ^*d*^NBS, aqueous acetate buffer (pH 4), RT, <1 min, 5–8%; ^*e*^NBA, acidic buffer (pH 4), RT, 15–20 min, 20–40%, ^*f*^NBS, 8 (M) aqueous urea, RT, 200 min, yield not reported; ^*g*^BNPS-skatole/AcOH, RT, 20–30 min, 15%; ^*h*^TBC/AcOH (pH 3), RT, 5–15 min, 50%; ^*i*^NCS/50–80% acetic acid (pH 4–5), RT, 30 min, 35–40%; ^*j*^NCS, 4.68 (M) urea, 27.5% acetic acid, RT, 30 min, 50%; ^*k*^NaIO_4_, acetate buffer (pH 5), 0 °C, followed by acidic hydrolysis at RT, 92–94% at W-7 & 17–22% at W-14 of SWM; ^*l*^OIBA, 80% acetic acid, RT, 30 min, 70–100%].

The first W-selective non-enzymatic cleavage of peptides and proteins was reported by Patchornik *et al.* in 1958.^35^ The investigators found that the treatment of 2–3 moles of NBS on glucagon, the hyperglycemic–glycogenolytic peptide from the pancreas, containing only one tryptophan among 29 amino acid residues, selectively ruptures the C-terminal tryptophyl bond in acetate–formate buffer at pH 4 (Fig. 2, path a). Based on the UV-spectral analysis of various indole and oxindole derivatives, they proposed the plausible mechanism for the reaction (for the general mechanism see Fig. 5Y also).^36^ In the presence of NBS, the indolyl side chain of the W-moiety 1 converts into oxindole derivative 2, which immediately hatches unstable iminolactone intermediate 5 (Fig. 2, path a). Hereon, we term this type of intermediate as a “Patchornik intermediate”. In acidic pH, the hydrolysis of the Patchornik intermediate furnished modified N-terminal peptide segment 7 containing spiro-2-oxindole-lactone moieties and intact C-terminal peptide fragment 9. Interestingly, the scission of glucagon by NBS is very rapid. Tryptophyl bond scission is completed within one minute. Besides, the reagent is more powerful than that of the known peptidases. Later, Ramachandran *et al.* applied this protocol for the W-selective cleavage of more complex peptides and proteins such as tobacco mosaic virus (TMV) protein, human serum albumin (HSA), bovine serum albumin (BSA) and lysozyme.^37^ Upon treatment with NBS, the proteins that contain more than one tryptophan residue, split into small peptides. However, lysozyme, bearing seven W-residues was cut in much lower yields. To replace NBS, the investigators also utilized *N*-bromoacetamide (NBA) and obtained the same result. Unfortunately, NBS/NBA-mediated oxidation is not suitable in the presence of other amino acid residues such as cysteine (C) and methionine (M) given that NBS/NBA can also modify the side chains of these amino acids. Besides, the selectivity of the reagent is hampered when tyrosinyl and histidinyl amide bonds are present in the peptide.

In 1964, Funatsu *et al.* introduced another mild oxidizing reagent, *N*-bromourea (prepared by adding NBS to aqueous urea), the reactivity of which is almost 200 times less than that of NBS (Fig. 2, path a).^38^ As a result, this reagent facilitates the cleavage of tryptophanyl peptide bonds without scissoring the tyrosinyl or histidinyl peptide bonds.

In 1970, the Omenn group reported the use of a sophisticated oxidizing agent, BNPS-skatole, a bromine adduct of 2-(2-nitrophenylsulfenyl)-3-methyl-3-indol, for the selective scissoring of the single tryptophan residue in the staphylococcal nuclease in 50–70% acetic acid (Fig. 2, path a).^39^ Later, the same reagent was utilized by Burnett *et al.* to split the A1 protein of bovine and human myelin.^40^ BNPS-skatole in 50% acetic acid was also applied by Prof. Angelo Fontana to scissor the W-selective sites of A1 encephalitogenic protein and horse heart cytochrome.^41^

Soon, the Patchornik group introduced another mild brominating reagent, 2,4,6-tribromo-4-methylcyclohexadieno (TBC), which can also cleave the tryptophanyl peptide bonds in protein in 65% acetic acid without scissoring the tyrosinyl or histidinyl peptide bonds (Fig. 2, path a).^42^ This reagent oxidizes methionine to methionine sulfoxide and cysteine to cysteic acid but never ruptures other peptide bonds.

In 1976, Shechter *et al.* cleaved the tryptophanyl peptide bonds utilizing 2 equiv. *N*-chlorosuccinimide (NCS) at pH 4–5 (Fig. 2, path b).^43^ Fortunately, all other peptide bonds are resistant to rupture by this reagent. Under these conditions, only the M- and C-residues are oxidized keeping the other amino acid residues intact. Later, horse heart cytochrome c was successfully fragmented by Lischwe *et al.* and Savige *et al.* by applying NCS/urea^44^ and DMSO/HX^45^ (X = Cl and Br) reagents, respectively (Fig. 2, path b). After investigating all these halogen-mediated reactions, it can be concluded that tryptophanyl peptide bond rupture occurs only when the peptide/protein attains the “Patchornik intermediate” and NCS in acetic acid is the most selective reagent for the cleavage of the tryptophanyl peptide bonds in proteins.

In 1967, M. Z. Atassi designed a protocol for the W-selective cleavage of sperm whale myoglobin (SWM) in acetate buffer at pH 5 (Fig. 2, path c).^46^ The protein was treated with sodium metaperiodate (NaIO_4_) at 0 °C for 7 h, which modifies the indole side chains of the W-residues into oxindole moieties that are structurally similar to the Patchornik intermediate. Upon stirring the oxidized apomyoglobin in 0.1 (N) HCI for 24 h at 25 °C, intact C-terminal peptide fragment 9 and modified N-terminal peptide segment 8 containing spiro-2-oxindole-lactone moieties were obtained. Sequencing of the fragmented peptides was accomplished, which revealed that the M- and Y-residues were modified during the reaction. The authors hoped that this procedure will be applicable to several biomolecules.

In 1977, Ozols *et al.* developed a methodology for the quantitative cleavage of the tryptophanyl peptide bonds in peptides and proteins with concentrated CNBr in the presence of anhydrous heptafluorobutyric acid (C_3_F_7_COOH).^47^ The investigators successfully applied their protocol for the quantitative W-selective cleavage of cytochrome b_5_. However, the selectivity of the reagent is hampered in the presence of M-residues.

In 1979, Mahoney and Hermodson claimed that *o*-iodosobenzoic acid (OIBA) is an effective and specific cleavage agent for tryptophanyl peptide linkages (Fig. 2, path-c).^48^ However, Wachter *et al.* proved that OIBA possesses lower selectivity than that reported by Mahoney and Hermodson because the selectivity of this reagent is disturbed in the presence of Y- and H-residues.^49^ Johnson *et al.*^50^ and the Fontana group^51^ confirmed the observation by Wachter *et al.* Later, Mahoney *et al.* improved their protocol by adding a scavenging agent of OIBA in the reaction mixture.^52^ Actually, excess OIBA cleaves the tyrosyl residues present in polypeptides. Thus, by adding *p*-cresol, the side reaction can be stopped. Truly, OIBA is a good oxidizing agent. It oxidizes the indol ring of the W-residue into an oxindole moiety, which approaches the Patchornik intermediate, and eventually cleave the tryptophanyl peptide bonds.

**Fig. 3:**
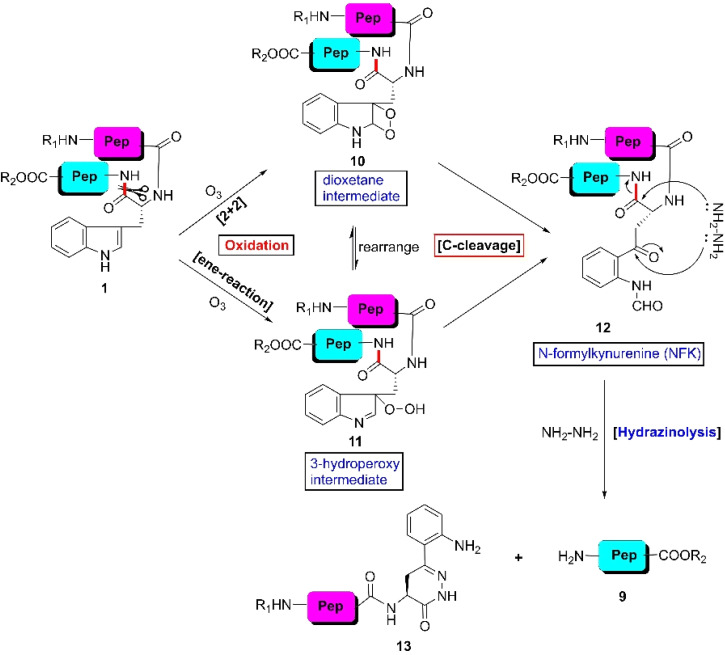
W-selective scissoring of peptides through NFK formation.

In 1966, Previero *et al.* ruptured C-terminal tryptophyl peptide bonds by passing a stream of ozone gas through a formic acid solution of tryptophyl peptide and resorcinol at 8–10 °C, followed by hydrolysis in strongly basic medium at 100 °C (Fig. 3).^53^ The reaction between the indole ring and ozone furnishes the *N*-formylkynurenine (NFK) intermediate 12 either through a dioxetane intermediate (10) or through 3-hydroperoxy intermediate (11). Upon treatment with NaHCO_3_–Na_2_CO_3_ at 100 °C, NFK ruptured into intact C-terminal peptide 9. However, the drawback of the protocol is that under these harsh conditions, denaturation of the protein can occur and other peptide bonds can also be cleaved. Thus, to avoid severe alkaline hydrolysis, Morishita *et al.* modified this protocol by treating NFK derivatives with hydrazine^54^ under much milder conditions to obtain N-terminal modified peptide segment 13 and C-terminal intact fragment 9 at RT. Fortunately, the modified protocol provided a better result for the cleavage of short peptides and W-bearing peptides of up to five residues were successfully cleaved at the specific site. However, both protocols were not tested for proteins or polypeptides.

**Scheme 1:**
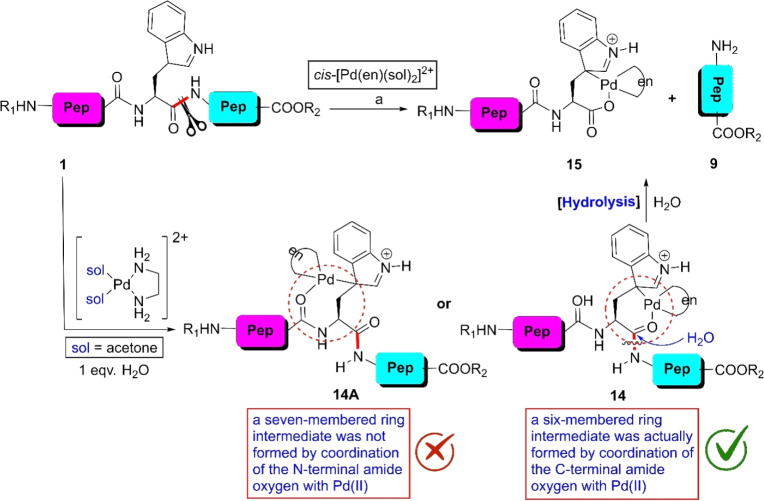
W-selective scissoring of peptides through metal complexation. Reaction conditions: (a) peptide : metal complex = 1 : 1, acetone-*d*_6_, DClO_4_, 50 °C. (This experiment was performed in an NMR tube).

Inspired by the coordinating ability of indole derivatives with metal ions in biological systems, the Kostic group developed an excellent protocol for the W-selective scissoring of peptides.^55^ When the Pd(ii)-complex *cis*-[Pd(en)(sol)_2_]^2+^ was mixed with peptides bearing a W-residue in acetone, complex 14 was formed exclusively (Scheme 1). The formation of 14 was guided by the stereochemical preference of the six-membered ring (14) over seven-membered cyclic moieties (14A). In this complex, the indole moiety exists in its tautomeric form and the amide carbonyl nicely coordinates with the metal ion. This coordination converts the amide carbonyl to more electrophilic. As a result, upon hydrolysis with water, the C-terminal side of the W-residue was fragmented into 9 and 15 avoiding the N-terminal end. The yield of the reaction depended on the amino acid present next to the W-residue. Amino acids containing a bulky side chain reduced the yield of the reaction. In this reaction, the solvent choice is also a significant issue. Water drastically inhibits the tryptophan coordination with Pd(ii), given that water itself can coordinate with the central metal ion. Besides, this methodology becomes vague if methionine and histidine residues are present in the peptide chain given that the coordinating ability of these amino acids with Pd(ii) is greater than tryptophan. Later, the investigators investigated this protocol applying Pt(ii)-complexes and obtained similar results.^56^

#### 3.1.2. Electrochemical method

**Scheme 2:**
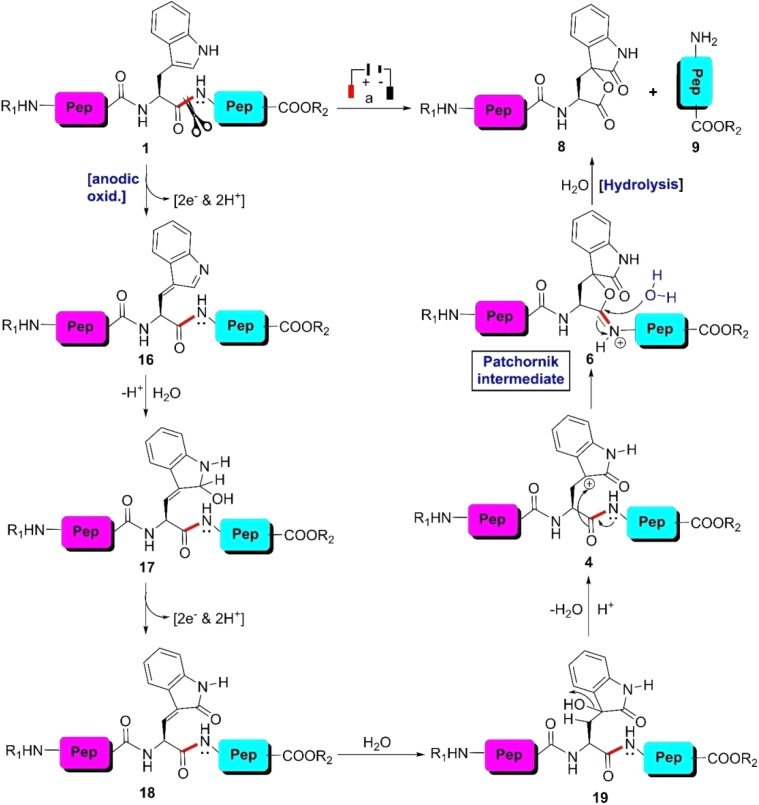
W-selective cleavage of peptide bond *via* electrochemical approach. Reaction conditions: (a) divided cell setup, graphite-anode, Pt-cathode, +1.5 V potential, Britton–Robinson buffer (pH 3).

Among the 20 common coded amino acids, side chains of cysteine, tyrosine, tryptophan, histidine, and methionine are electrochemically oxidizable at graphite electrodes.^57–61^ Employing the benefit of this electrochemical activity of the W-residue, MacDonald *et al.*, for the first time, fragmented W-tagged dipeptides at a potential in the range of 1.8–2.2 V (*vs.* SCE) applying Pt-electrodes in phosphate buffer solution (pH 7).^62^ Soon after, this protocol was accelerated by the Walton group, who ruptured the selective-site of hen egg-white lysozyme (HEWL), a small protein bearing 129 amino acids, under electrochemical conditions.^63^ HEWL was electrolyzed between a graphite anode and a platinum cathode in Britton–Robinson buffer at a potential above +1.5 V until new polypeptides with a lower molar mass were obtained (Scheme 2). The fragments were sequenced using a biosystems 470A gas phase sequencer, which revealed that HEWL was cleaved between W-62 and W-63, producing two fragments with a relative molar mass (RMM) of 6.9 and 7.4 kDa, respectively.

In the case of electrochemical cleavage of W-bearing peptides, the reaction starts with the anodic oxidation of the indole moiety (1) in acetonitrile–water (1 : 1) solvent in the presence of 0.1% formic acid (pH 2.8) to generate oxindole intermediate 19 through several reactive species (Scheme 2). The reaction proceeded in a divided cell setup consisting of a graphite-anode and a Pt-cathode, applying a potential of +1.5 V. Next, 19 was transformed into the Patchornik intermediate (6) involving the downstream amide bond. Ultimately, activation of the amide bond furnished fragment 8 and fragment 9 through hydrolysis. It was observed that the efficiency of the reaction was the maximum at pH 3.

Several larger peptides and proteins of various sizes and compositions such as neuropeptide Y, insulin, RNase I, α-lactalbumin, apomyoglobin, and lysozyme were site-selectively cleaved as well as analyzed by Permentier and Bruins utilizing online electrochemistry-mass spectrometry (EC-MS) technology.^64^ The oligopeptides/proteins dissolved in water–acetonitrile–formic acid solutions (pH 2.8) were passed through a syringe pump at a flow rate of 2 μL min^−1^ connected with a Coulochem 5021 conditioning cell containing a porous graphite working electrode, a palladium auxiliary electrode, and a palladium reference electrode. The cell potential was varied from 0.8 to 1.2 V. The oxidized products analyzed by offline liquid chromatography-mass spectrometry (LC-MS) experiments ensured that W-selective peptide bond cleavage occurred successfully. Applying this protocol, a series of tripeptides consisting of W-residues (LWL, GWG, EWE, and KWK) were site-selectively cleaved as well as analyzed by the Bischoff group.^65^ Unlike the experiments reported by Permentier,^64,66^ these investigators scanned several adjacent amino acids with respect to the W-residue to verify the effect of size and charge on cleaving efficiency of the tripeptides. The effects of pH and electrolyte on the scissoring efficiency were also checked. Unstable reaction products were detected by online EC-MS, while isobaric oxidation products were separated by LC experiments. The LC-(MS/MS) technique accurately determined the yields of the reaction and product distribution. Based on this information, the authors proposed the reaction mechanism, as shown in Scheme 2. The advantage of this protocol is that the cleaving technique is fully instrumental. Thus, it has the potential for full automation. Moreover, the speed of the analysis is very rapid and the Y-selective peptide bond can also be ruptured by simply tuning the potential range. However, this protocol provides lower yields compared to enzymatic and chemical methods due to the competing side-chain oxidation reactions.

#### 3.1.3. Chemoenzymatic method

**Scheme 3:**
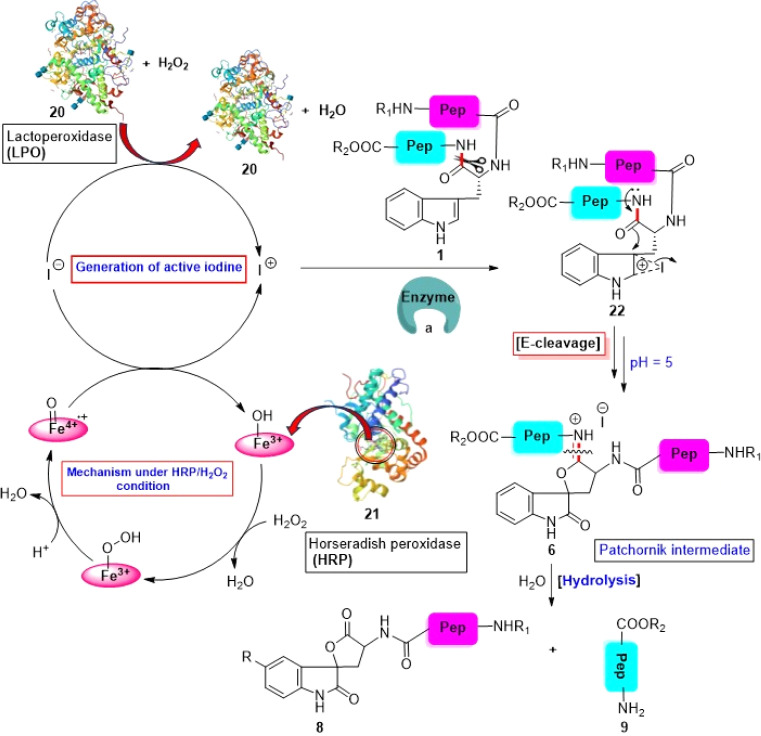
W-selective cleavage of peptide bond *via* chemoenzymatic approach. Reaction conditions: (a) enzyme (LPO or HRP), KI, H_2_O_2_, acetate buffer (pH 5), RT, 10 min, 30–40%.

According to the mechanistic details of bromination of W-tagged peptides proposed by Patchornik *et al.*,^36^ an excellent chemoenzymatic tryptophanyl peptide bond scissoring protocol was developed by N. M. Alexander.^67,68^ Short peptides bearing W-residues were treated with KI and H_2_O_2_ in the presence of either lactoperoxidase (LPO, 20) or horseradish peroxidase (HRP, 21) at 23 °C. HRP is a large alpha-helical glycoprotein that binds heme as a redox cofactor. In the presence of H_2_O_2_, its heme part participates in the catalytic cycle and liberates “active iodine” from KI (Scheme 3) in acetate buffer (pH 5) at RT. Likewise, a mixture of LPO and H_2_O_2_ can convert iodide into “active iodine” in acetate buffer (pH 5) at RT (Scheme 3). This “active iodine” converts peptide 1 into reactive intermediate 22. Then, chemoenzymatically produced species 22 is transformed into the Patchornik intermediate (6) through the activation of the downstream amide bond (with respect to the indolyl side chain). Ultimately, *in situ* hydrolysis of 6 furnishes modified N-terminal peptide segment 8 and intact C-terminal peptide fragment 9, as described in Section 3.1.1. The maximum LPO-catalyzed fission occurs at pH 4–6, while in the case of the HRP-catalyzed reaction, it occurs at 4–5. It has been found that LPO is 2.5 times more efficient on a weight basis than HRP. Competition experiments with equimolar amounts of different W- and Y-tagged dipeptides revealed that W-oxidation proceeded selectively at pH 5. The most important feature of enzyme-catalyzed tryptophanyl peptide bond scissoring is that it may induce the intracellular hydrolysis of thyroglobulin, an inevitable step for the secretion of thyroid hormones from the thyroid into the blood plasma for transport to the peripheral tissues. Therefore, in the future, this protocol may be utilized for therapeutic purposes. However, the yield of the reaction is quite low. LPO can cleave only 37% of the peptide after 10 min of the reaction and the yield can be increased up to 50% by doubling the H_2_O_2_ and iodide concentrations.

In conclusion, in Section 3.1, the most commonly employed chemical protocol for the cleavage of the tryptophanyl peptide bond is chemical cleavage using OIBA due to its high yield and specificity compared to other methods.

### 3.2. Tyrosine

The tyrosine residue possesses a phenolic side chain consisting of some special features. Firstly, its aromatic ring is electron-rich, which promotes the tyrosine-selective sites of peptides to be ruptured by chemical approaches. Secondly, the redox-active behavior of the phenolic moiety provides an opportunity to scissor the tyrosyl-peptide bonds by employing sophisticated electrochemical technologies. Recently, an enzyme has been discovered that can lacerate the tyrosine-tagged sites by chemoenzymatic techniques. The strategies that initiated the Y-selective cleavage of peptide bonds in peptides are showcased.

#### 3.2.1. Chemical method

**Scheme 4:**
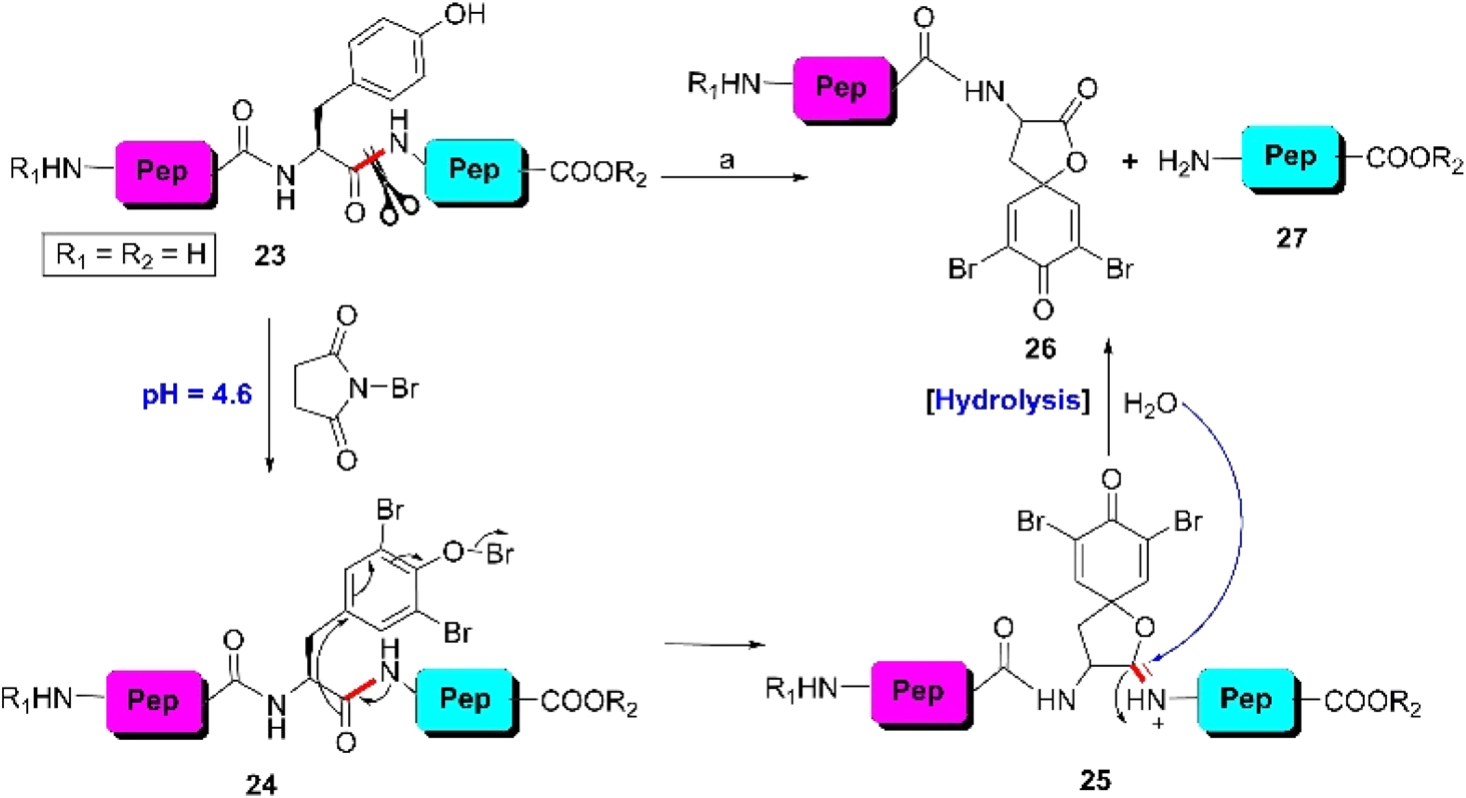
Y-selective cleavage of the peptide bond through oxidative bromination. Reaction conditions: (a) NBS, 20% acetonitrile–acetate buffer (pH 4.6), RT, 10–30 min, 40–50%.

The first site-selective cleavage of peptides and proteins at the Y-residue was reported by Schmir *et al.* in 1959 through the bromination of native peptides by NBS at RT.^69^ This reaction was initiated through the oxidative bromination of peptide 23 to obtain tribromo-product 24 at pH 4.6. Then, the immediate formation of hydrolysable carboximidate intermediate 25, which is structurally similar to the Patchornik intermediate, cleaved the C-terminal amide of tyrosine to provide intact C-terminal peptide fragment 27 and modified N-terminal peptide segment 26 containing a spirodienone-lactone moiety (Scheme 4). This reaction exhibited a wide substrate scope, encompassing both peptides and proteins.^70–72^ However, this protocol is not acceptable in the presence of other amino acid residues such as M-, C-, and H-residues given that the NBS-mediated reaction can alter the side chains of these amino acids. More importantly, in the case of peptides and proteins containing both Y- and W-residues, this methodology provides better results by cleaving the tryptophyl peptide bonds (see Section 3.1.1). However, NBS-mediated cleavage of the tryptophanyl peptide can be prevented by converting it into *N*-formylkynurenine by passing a stream of ozone gas through the peptide solution containing formic acid and resorcinol (Fig. 3).

In 1969 Junek *et al.* replaced NBS with *N*-iodosuccinimide (NIS) and obtained similar results.^73^

**Scheme 5:**
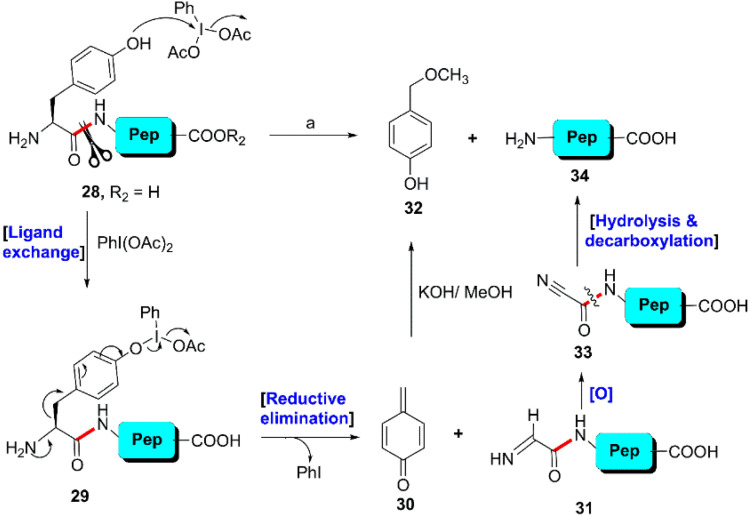
Y-selective cleavage of the peptide bond through oxidation using hypervalent iodine. Reaction conditions: (a) PhI(OAc)_2_, KOH–MeOH, 0–5 °C, 1.5 h, 60–80%.

In the mid-80's, Moriarty *et al.* observed that among the 20 proteinogenic amino acids, tyrosine selectively reacts with (diacetoxyiodo)benzene in the presence of alcoholic KOH to yield cyanomethylphenol (Scheme 5). Applying this reaction, the investigators tactfully cleaved a series of unprotected dipeptides (28) containing an N-terminal Y-residue under mild conditions.^74^ The reaction was initiated with the coordination of the hydroxyl group of the Y-residue with the iodine(iii) center. Then, reductive elimination of phenyl iodide from intermediate 29 produced reactive species 4-methylenecyclohexadienone (30) and 31. Immediately, 30 transformed into 4-(methoxymethyl)phenol (32) in the presence of alkaline MeOH and 31 was converted into the corresponding C-terminal amino acid (34) through oxidation followed by immediate hydrolysis and decarboxylation (Scheme 5). However, due to the limited substrate scope for N-terminal tyrosyl peptides, no additional studies or applications of this reaction have been reported thus far.

**Scheme 6:**
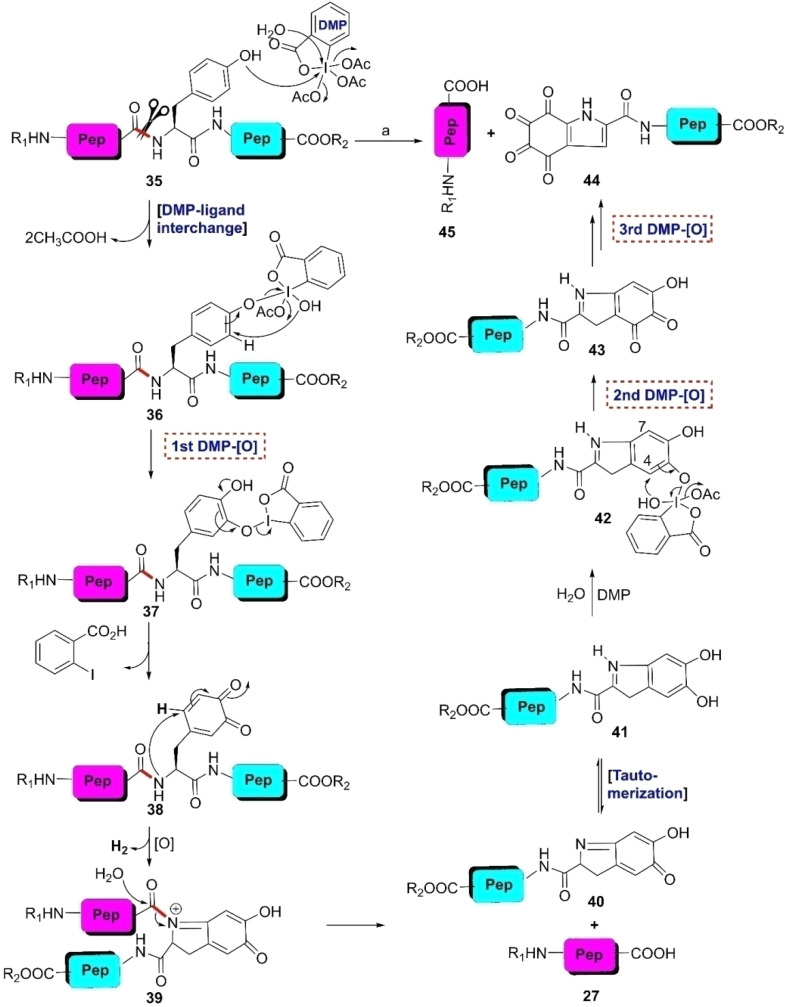
Y-selective cleavage of the peptide bond through DMP-mediated oxidation. Reaction conditions: (a) DMP (excess), DMSO, phosphate buffer (pH 7), 40 °C, 2–3 h.

Recently, the Brimble group discovered that Dess–Martin periodinane (DMP) is an excellent reagent for the selective scission of the N-terminal amide bond of Y-residues in a mixture of DMSO and PBS buffer in a neutral medium (pH 7.0) at 40 °C.^75^ The rupture of the tyrosinyl peptide bond by DMP provided C-terminal peptide fragment 44 bearing an unusual hyperoxidized tyrosine moiety, 4,5,6,7-tetraoxo-1*H*-indole-2-carboxamide (TICA), together with unaltered N-terminal peptide fragment 45 (Scheme 6). Compared to the other Y-selective peptide bond cleavage protocols, it exhibited high selectivity for Y-residues together with high functional group tolerance of natural and diverse unnatural amino acids. During DMP-mediated oxidation, except for Cys, side chains of Trp, Ser, Thr, *etc.* remained unaffected. However, free peptide N-terminal amine impedes the cleavage of the tyrosinyl peptide bond. As a result, protection of N-terminal amine by the acetyl group becomes an unavoidable job before executing the DMP-mediated reaction. Applying this protocol, oligopeptides with up to 30 amino acid residues were modified easily. Besides, using DMP, naturally occurring cyclic peptides such as dichotomin J (isolated from *Stellaria dichotoma*^76^), szentiamide (isolated from *Xenorhabdus szentirmaii*^77^), and antifungal cyclic lipopeptide iturin A^78^ were successfully ruptured as well as linearized at the Y-selective sites. The linearized peptides were identified by the MS/MS technique. An interesting fact is that TICA is highly electrophilic in nature. Thus, DMP-modified peptides (44) can be further altered through selective bioconjugation and synthetic manipulation.

#### 3.2.2. Electrochemical method

**Scheme 7:**
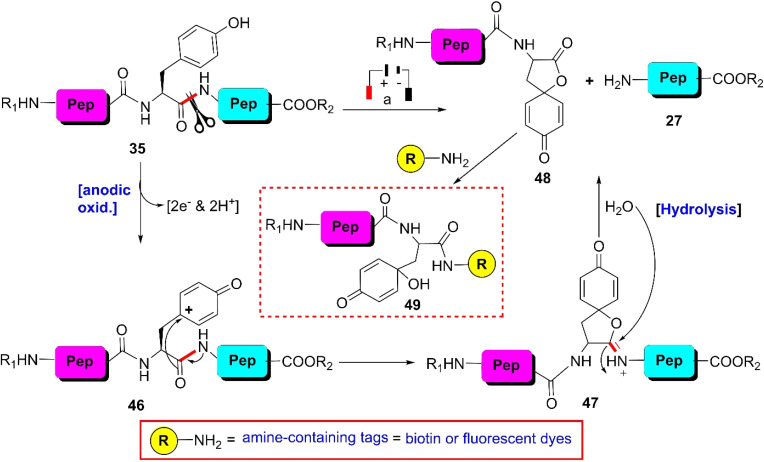
Y-selective cleavage of peptide bond *via* electrochemical approach. Reaction conditions: (a) porous graphite electrodes (*vs.* Pd reference electrode), constant potential (0.8 V or 1.0 V), acetonitrile–water–formic acid (pH 2.8), flow rate 2 μL min^−1^.

Inspired by the seminal work of Iwasaki *et al.*^79,80^ and the Cohen group^81,82^ published in the 1960s, which detailed the electrochemical cleavage of tyrosyl peptide bonds applying a divided cell setup and continuous flow chemistry, the Bruins group revealed that electrochemical oxidation of Y- and W-residues within peptides leads to specific cleavage of the amide bond at their C-terminal side.^66^ Earlier electrochemical approaches were incompatible due to the boiling of the proteins. Upon electrochemical oxidation of the phenol group, phenoxonium group 46 is generated. Subsequently, this intermediate can undergo lactonization with the carbonyl group of the C-terminal amide to give intermediate 47, which upon hydrolysis, yields an N-terminal peptide fragment bearing spirodienone-lactone unit 48 and unmodified C-terminal peptide 27 (Scheme 7). Achieving site-selectivity of Y- over W-residue is challenging due to their similar oxidation potential in acidic solutions, which are the optimal conditions to achieve the cleavage reaction.^83^ M- and C-residues were also oxidized during this process, but disulfide bonds remained intact.

A wide variety of substrates, ranging from tripeptides to proteins, have been successfully cleaved at the Y- and W-sites in electrochemical cells by just tuning the potential of the cell.^64,65^ The most remarkable feature of this cleavage method is its compatibility with MS and LC-MS analysis. The electrochemical cell can be directly coupled to a mass spectrometer, thereby allowing fast and real-time analysis of the complex protein digest without the need for extra sample preparation. However, the electrochemical oxidation of proteins and peptides suffered from inherent low yields given that a mixture of non-cleavable oxidation products was generated simultaneously during the cleavage. It is also interesting to note that the generated spirolactone moiety of cleaved N-terminal fragment 47 is susceptible to nucleophilic attack. Aminolysis of the spirolactone with several amine-containing tags, such as biotin and fluorescent dyes, would afford compound 49, enabling the selective labeling and enrichment of electrochemically cleaved peptides (Scheme 7).^84^

#### 3.2.3. Chemoenzymatic method

**Scheme 8:**
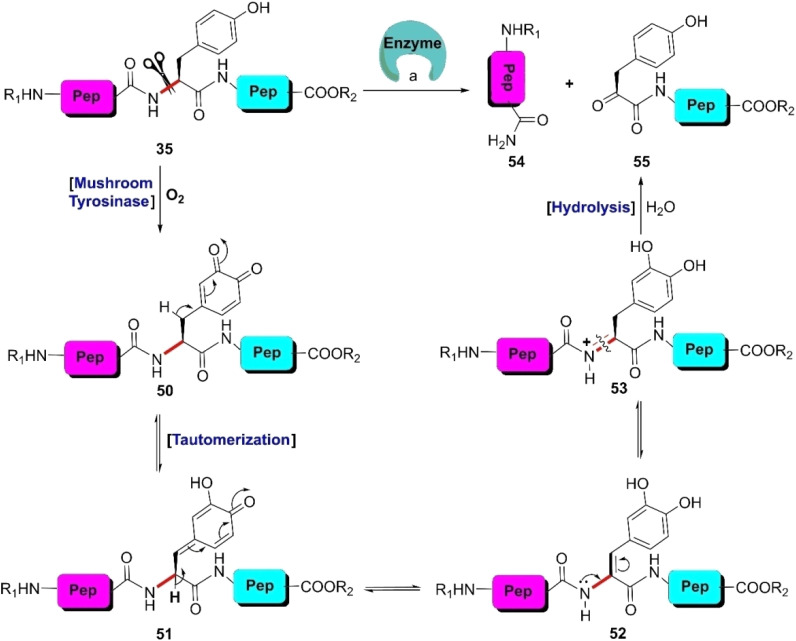
Y-selective cleavage of peptide bond *via* chemoenzymatic approach. Reaction conditions: (a) mushroom tyrosinase, phosphate buffer (pH 6.6), 37 °C.

Enzymatic hydrolysis of the tyrosinyl peptide bond is very rare. In 2012, Hedstrom and colleague identified an enzyme called ‘mushroom tyrosinase’, which has the potential to catalyze this type of transformation. According to their findings, unstructured tyrosine-rich sequences such as hemagglutinin (HA) tags (YPYDVPDYA) attached to *Escherichia coli* dihydrofolate reductase were selectively cleaved at the N-terminal amide of tyrosine by mushroom tyrosinase in phosphate buffer (pH 6.6) at 37 °C *via ortho*-quinone intermediate 50 (Scheme 8).^85^ Probably this quinone intermediate undergoes a series of tautomerizations to regain the aromatization. Thus, acyl enamine intermediate 52 is formed, which is converted into acyl enamine 53 by abstracting a proton from the reaction environment. 53 is labile to hydrolysis under aqueous conditions to afford N-terminal peptide amide 54 and C-terminal α-keto amide 55. This reaction is beneficial in low protein concentrations and in the absence of exogenous nucleophiles. Under these conditions, *o*-quinone decomposes with fragmentation of the HA tag furnishing the products. However, at higher protein concentrations (>5 mg mL^−1^), crosslinking is favoured. However, the presence of exogenous nucleophiles and a high protein concentration disfavors this process owing to interception of the generated *ortho*-quinone and potential protein cross-linking.

Besides mushroom tyrosinase, some enzymes present in peptides and proteins can selectively cleave engineered tyrosines such as phosphotyrosine, *o*-sulfated tyrosine, and 3-nitrotyrosine. In 2007, Knight *et al.* identified a subtilisin variant (E156R/P129G) that selectively cleaves phosphotyrosine peptides.^86^ Alternatively, Varadarajan *et al.* discovered two *E. coli* outer membrane protease (OmpT) variants that preferably rupture the sulfotyrosine residues in peptide substrates.^87^ Later, the same research group isolated a highly active variant of the *E. coli* endopeptidase OmpT that selectively hydrolyzes peptides after 3-nitrotyrosine.^88^

### 3.3. Phenylalanine

Phenylalanine (Phe or F) is an essential amino acid, which is introduced into protein molecules by encoding the codons UUU and UUC. It contains a benzyl side chain to introduce hydrophobicity into the molecule. F-residues play a crucial role in dictating the structure and folding of proteins. Furthermore, it is the precursor of many important biomolecules such as tyrosine, the neurotransmitter dopamine, and norepinephrine. Thus, F-selective cleavage by means of various chemical and chemoenzymatic techniques followed by analysis of the protein fragments may give in-depth information on proteins. Unfortunately, phenylalanine is a redox-inactive amino acid. Thus, there is no possibility for electrochemical cleavage of peptides at F-residues. Here, we describe the strategies that promoted the F-selective cleavage of peptide bonds in peptides and proteins.

#### 3.3.1. Chemical method

**Scheme 9:**
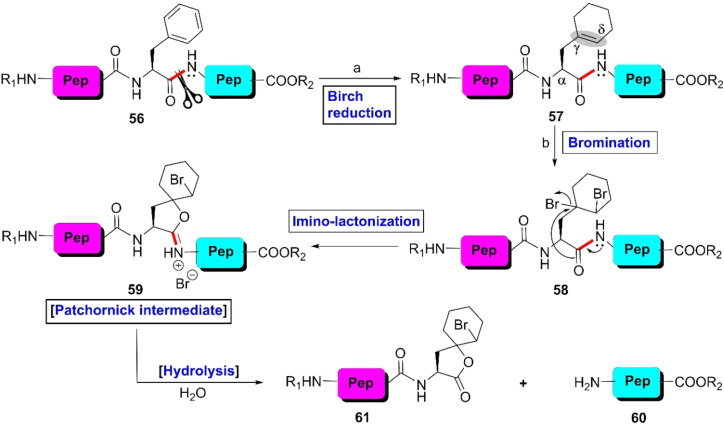
F-selective cleavage of peptide bond *via* the Birch reaction. Reaction conditions: (a) Li, CH_3_NH_2_, −70 °C, 2–3 h. (b) Br_2_/AcOH or NBS/AcOH.

The first site-selective non-enzymatic cleavage of F-residues in peptides was developed by Wilchek *et al.* in 1962.^89^ According to the previous reports on the NBS-mediated oxidative cleavage of tryptophanyl and tyrosinyl peptide linkages, these investigators guessed that a γ–δ double bond with respect to the carbonyl moiety is essential for the successful cleavage of peptide bonds. Fortunately, native peptides bearing an F-residue contain a γ–δ double bond with respect to the carbonyl moiety. Thus, they attempted to scissor phenylalanyl peptide bonds through NBS-promoted oxidation at RT. Unfortunately, they failed due to the lower reactivity of the phenyl ring towards NBS. To improve the reactivity profile, the investigators tactfully reduced the phenyl ring into cyclohexenyl system 57 through Birch reduction^90^ and could obtain Patchornik intermediate 59 by the NBS-mediated oxidation of 57 in 50% acetic acid. Then, hydrolysis of 59 furnished intact C-terminal peptide-fragment 60 and modified N-terminal peptide-segment 61 containing spiro-2-bromocyclohexane-lactone moieties (Scheme 9). However, other amino acid residues and protecting groups that contain aromatic rings also undergo Birch reduction and interfere with the reaction. Moreover, this protocol is not suitable for oligopeptides and proteins bearing versatile functionalities.

**Scheme 10:**
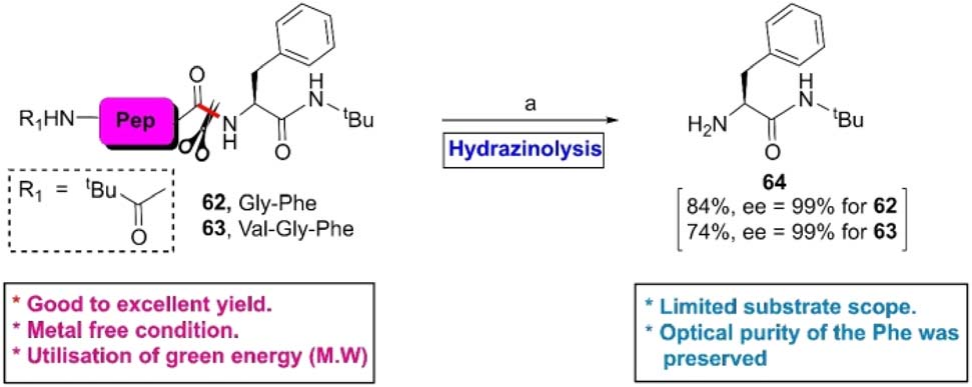
Y-selective cleavage of peptide bond *via* hydrazinolysis. Reaction conditions: (a) NH_2_NH_2_·H_2_O, NH_4_I, C_2_H_5_OH, and microwave heating at 70 °C until the release of the F-residue.

As an extension of their previous work,^91^ in 2014, Shimizu *et al.* accomplished the hydrazinolysis of several di- and tri-peptides in the presence of ammonium salt at 70 °C under microwave (MW) conditions.^92^ After the reaction, it was observed that the N-terminal peptide bond of the F-residue (62 or 63) was selectively fragmented to 64 in good to excellent yields without any epimerization of the α-position of the ruptured amino acids (Scheme 10). The authors assumed that the yield of the reaction and site-selectivity were directed by both steric and electronic factors.

#### 3.3.2. Electrochemical method

**Fig. 4:**
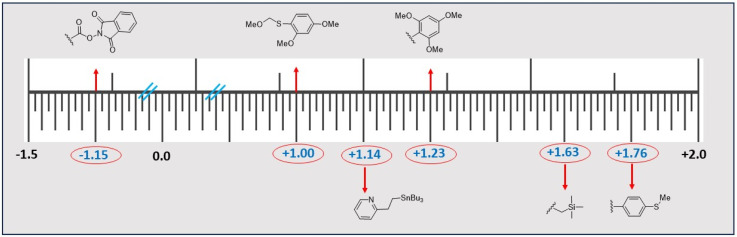
Popular electro-auxiliaries and their corresponding activation potentials (in voltage *vs.* Ag/AgCl).

The seminal work of Brabec *et al.* explored that at graphite electrodes, only the side chains of tryptophan, tyrosine, histidine, cysteine and methionine are electrochemically oxidizable.^93^ Naturally, employing electrochemistry it is impossible to scissor the F-selective sites of unmodified peptides and proteins. However, by installing suitable electroauxiliaries (some of these electroauxiliaries and their corresponding activation potentials (*vs.* Ag/AgCl)^94–100^ are shown in Fig. 4) into the F-selective sites one can make them redox active. Thereafter, benefitting from this redox activity, perhaps phenylalaninyl peptide bond rupture may be possible. However, this type of approach has not been documented to date.

#### 3.3.3. Chemoenzymatic method

**Scheme 11:**
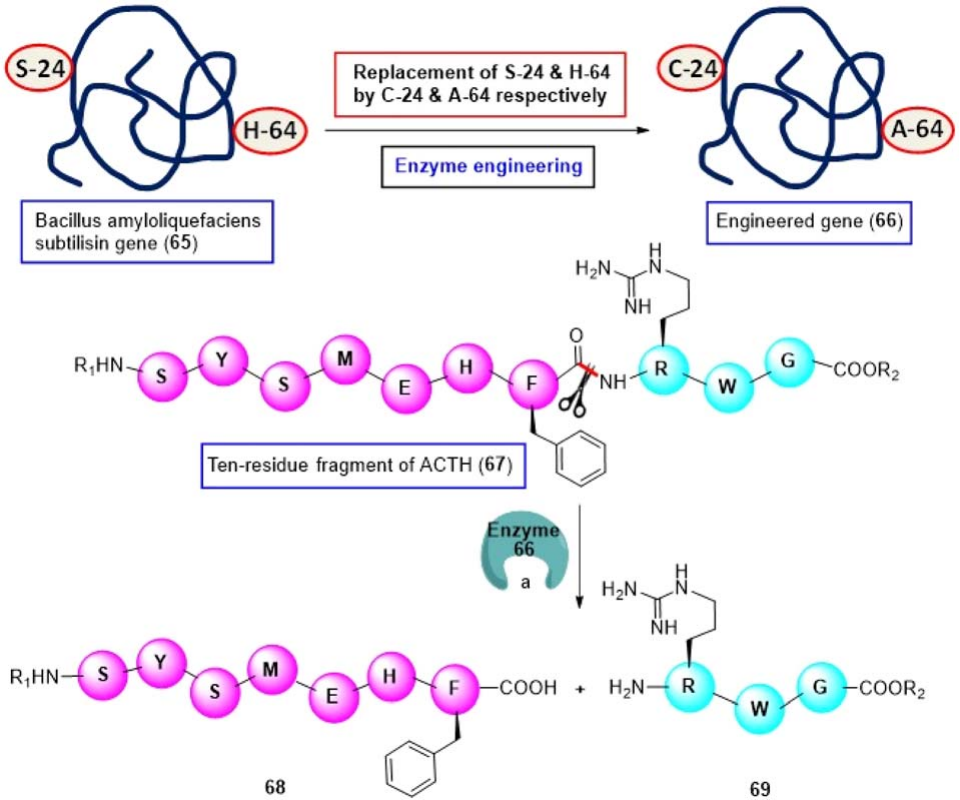
F-selective cleavage of peptide bond *via* chemoenzymatic approach. Reaction conditions: (a) S24C:H64A, Tris–HCI (pH 8.0), dithiothreitol (DTT), dimethylsulfoxide (DMSO), phenylmethylsulfonyl fluoride (PMSF), 37 °C, 20 h.

It was observed that the aromatic side chain of phenylalanine and tyrosine of some specific peptides can fit into the binding pocket of several wild-type subtilisin BPN enzymes. Consequently, the N-terminus of the scissile bond of the substrate can come in contact with the active site of the enzyme. As a result, these enzymes can hydrolyze the peptide linkage of these specific peptides.^86^ However, Carter and Wells were interested in creating new mutants of these wild-type subtilisins. Thus, in 1987, they tactfully replaced the serine 24 (S24) and H64 residues of the *Bacillus amyloliquefaciens subtilisin* gene (65) with cysteine (C) and alanine (A) residues, respectively, (this mutant is abbreviated as S24C:H64A, Scheme 11), and studied the catalytic efficiency of this engineered enzyme (66).^101^

The investigators digested a ten-residue fragment (SYSMEHFRWG, 67) of human adrenocorticotropic hormone (ACTH) with S24C:H64A at 37 °C in the presence of Tris–HCI (pH 8.0), dithiothreitol (DTT), dimethyl sulfoxide (DMSO) and phenylmethylsulfonyl fluoride (PMSF) for 20 h and observed that the C-terminal peptide linkage of the F-residue was fragmented quantitatively to afford peptide fragments 68 and 69 (Scheme 11). Their further studies revealed that this bond rupturing is guided by the upstream H-residue present in the peptide. The same engineered enzyme was utilized to cleave the C-terminal peptide bond of a Y-residue present in the 20-residue fragment of the inhibin β chain.^101^

### 3.4. Histidine

Histidine (His or H) is an essential amino acid, which is inserted into protein molecules by encoding the codons CAU and CCA. It is an integral part of the catalytic mechanism of many enzymes. Histidine plays a crucial role in biological processes such as catalytic triads and proton shuttle mechanism. The imidazole moiety of the H-residue commonly serves as a ligand in metalloproteins. Naturally, cleavage at the H-selective site of peptides and proteins is very important. The protocols that enabled the H-selective cleavage of peptide bonds in peptides/proteins are detailed below.

#### 3.4.1. Chemical method

**Fig. 5:**
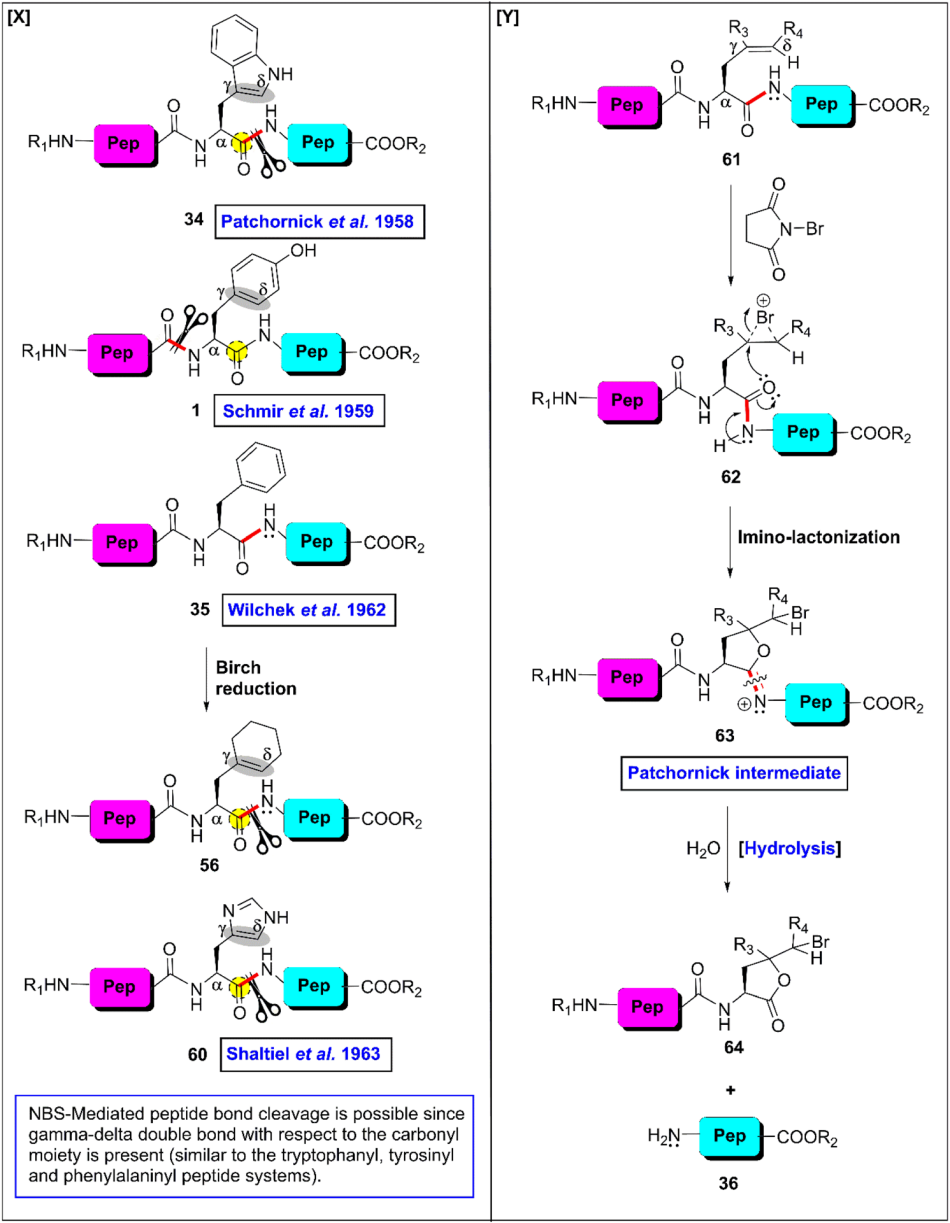
NBS-enabled cleavage of residue-selective peptide bonds. (X) Different substrates (Y) general mechanism.

The first site-selective non-enzymatic cleavage of peptides at the H-residue was reported by Shaltiel *et al.* taking the advantage of the γ–δ double bond (of the imidazole ring) with respect to the carbonyl moiety.^102^ Actually, in the presence of NBS, the γ–δ double bond (71) of the peptide side chain is converted into the corresponding cyclic bromonium cationic intermediate (72), which is converted into the Patchornik intermediate (73) through the imino-lactonization process. Finally, hydrolysis of 73 produces two specific peptide fragments, 74 and 9. Considering this mechanism, the NBS-mediated oxidative technique was employed to oxidize the imidazole ring of the H-residues in pyridine acetate buffer (pH 3–4) at RT. After that, in the presence of excess NBS, the reaction mixture was refluxed at 100 °C for 1 h (Fig. 5Y provides the general mechanism). The hydrolysis successfully fragmented H-bearing dipeptides with yields of 50–65%. However, the scissoring efficiency significantly decreased for polypeptides bearing 5–10 amino acids. Moreover, cleavage of the tryptophyl and tyrosyl peptide bonds could not be avoided. However, they could be differentiated. The reaction between NBS and peptide containing W-, Y- and H-residues at RT in pyridine-acetic acid-water (1 : 10 : 19 v/v) split the tryptophyl and tyrosyl linkages only. Under these conditions, the histidyl residues were oxidized but not cleaved. After destroying the excess NBS and heating the reaction mixture, the histidyl peptides were fragmented.

**Scheme 12:**
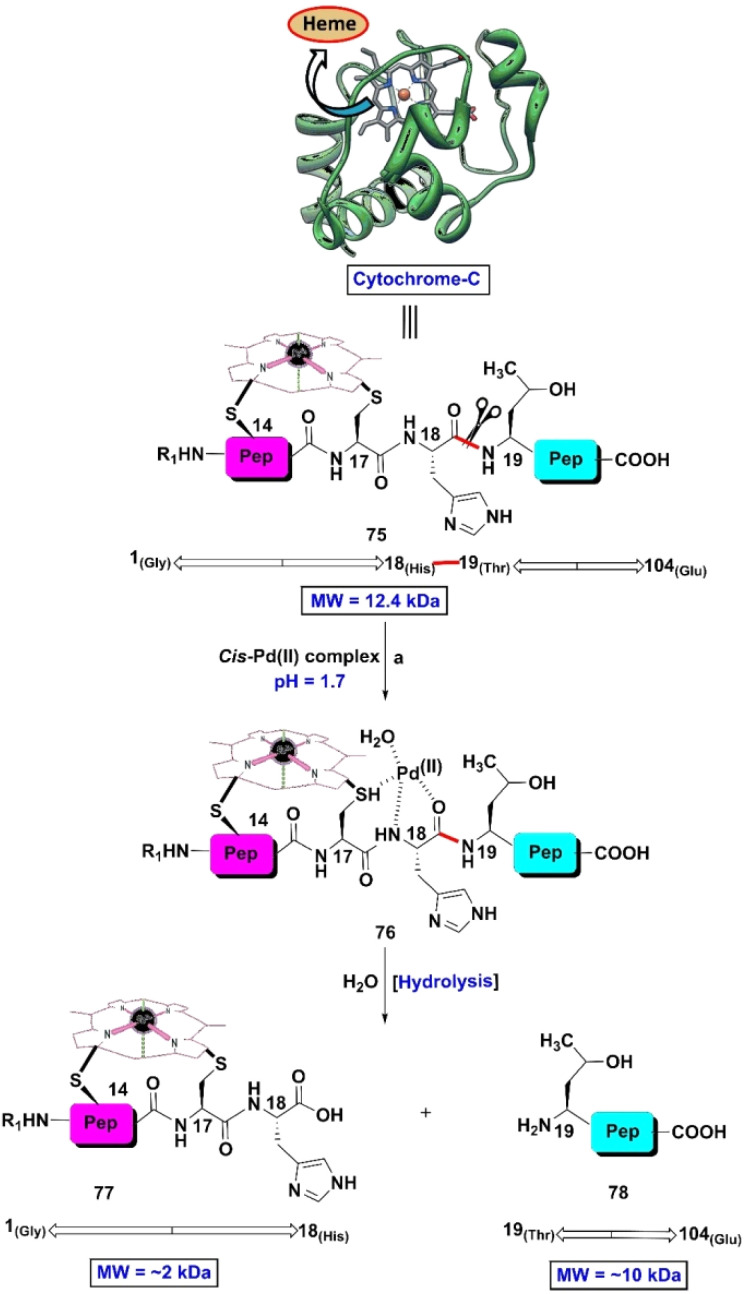
Site-selective cleavage of cytochrome C *via* Pd(ii) complexation. Reaction conditions: (a) acidic promoter (HBF_4_, HCIO_4_ or CF_3_COOH), 40 °C, 2 days.

Utilizing the coordinating ability of H-residues (through the imidazolyl side chain) with selective metal ions, Mother Nature synthesizes several metalloproteins. Learning from this lesson in nature, in the 1990s, scientists accepted the challenge of synthesizing different metal complexes that can act as artificial peptidases. To the best of our knowledge, Prof. Kostic and colleagues were the first group of investigators who developed four Pd(ii)-complexes (utilizing water, diethylenediamine, 1,5-dithiacyclooctane and its 3-hydroxy derivative as ligands) that could scissor the selective peptide bond of cytochrome c (75), a hemeprotein found in the inner membrane of the mitochondrion.^103^ The protein in a solution containing Pd(ii)-complex was treated with a non-coordinating acid such as HBF_4_, HCIO_4_ or CF_3_COOH, causing it to exhibit the partially unfolded state and come closer to the Pd(ii)-center to furnish complex 76. This aliquot (at pH 1.4) was incubated for 2 days at 40 °C and it was confirmed through various experiments that the peptide linkage between histidine 18–threonine 19 (H18–T19) was ruptured site-selectively to provide fragments 77 and 78 with RMM (approx.) of 2 kDa and 10 kDa respectively (Scheme 12). Cytochrome c consists of 104 residues, in which position-18, 26 and 33 are occupied by H-residues. However, the C-terminal peptide linkage of H18 (H18–T19) was fragmented selectively, avoiding the other H-connected peptide bonds. This site selectivity was achieved due to the presence of a cysteine residue (C17) just before H18. This C17 invites the metal complex to bind at that particular position and scissor the H18–T19 peptide linkage. In cytochrome c, there is another cysteine at position 14, after which there is an alanine residue (A15, bearing a non-coordinating methyl side chain). The C-residue has sufficient potential to co-ordinate with metal ions; however, in the sterically hindered protein environment, C14 alone cannot hold the metal ion. As a result, the peptide bond next to A15 is never fragmented. Thus, in the C17-directed fragmentation process, the imidazole side chain of H18 plays a vital role. To reveal the role of T19, in the fragmentation process, a model tripeptide (C–H–T) was selected and the threonine residue was replaced by alanine (to obtain tripeptide C–H–A) and glycine (to obtain tripeptide C–H–A) residues. It was observed that in all cases, the H–A and H–G bonds were split. However, in the presence of *cis*-[Pd(en)(H_2_O)_2_]^2+^, another model segment of cytochrome c C(Me)14–A15–Q16 (Q: glutamine), the C(Me)–A peptide bond was ruptured, and cleavage of the A–Q linkage was never observed. In this case, cysteine alone can hold the metal ion and rupture the scissile bond. This proves that in the fragmentation process, H19 does not play any significant role; rather, the coordinating ability of the C and H-residues is important.

Based on the experimental and theoretical results obtained by B3LYP^104–106^ density functional theory in the Gaussian98 program package using the 3-21G, 6-31G(d) and LanL2DZ basis sets, Zhu and co-workers proposed the mechanism of H18–T19 peptide bond scissoring in cytochrome c.^107^ During the cleavage process, Pd(ii) coordinates with the carbonyl oxygen of H18, and simultaneously the hydrogen bond between the carbonyl oxygen and ancillary dimer water weakens as well as polarizes the C=O double bond of the H-residue. As a result, the H18–T19 peptide bond ruptures site selectively.

**Fig. 6:**
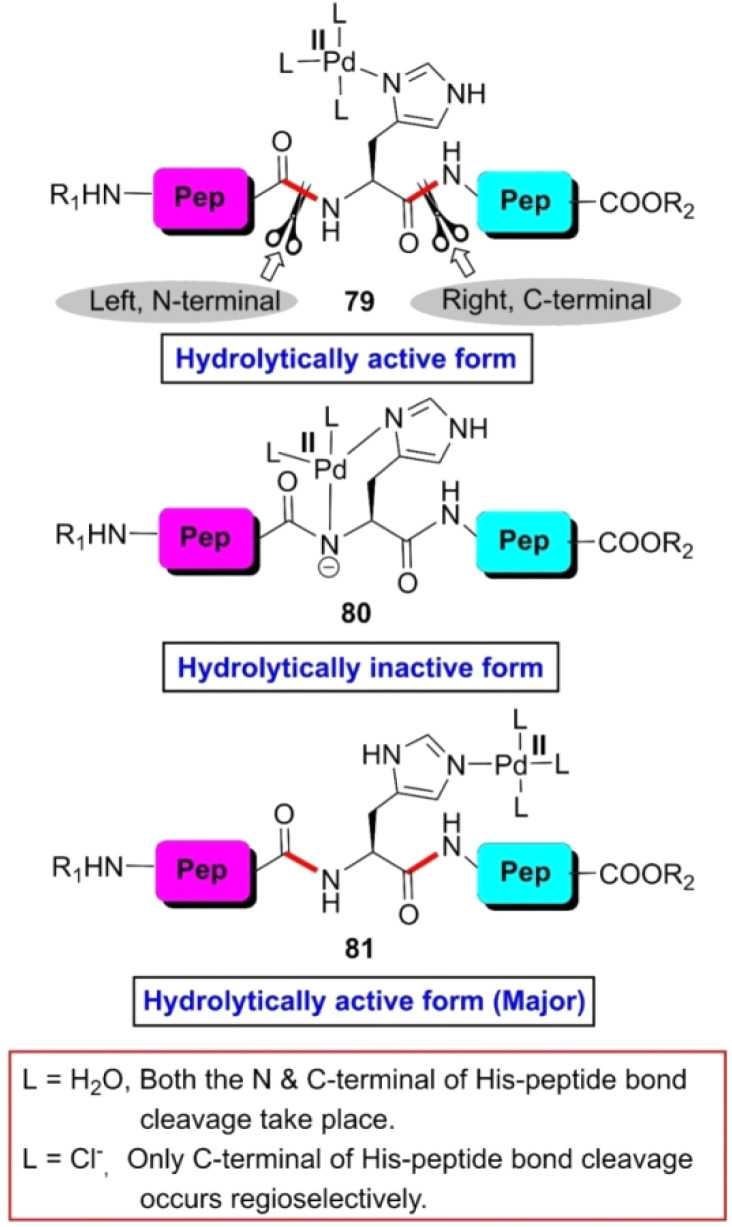
Modes of co-ordination of Pd(ii)-complex with histidinyl peptide. Reaction conditions: (a) histidinyl peptide, *cis*-[Pd(en)(H_2_O)_2_]^2+^, HBF_4_ (promoter), pH 1.5–2 (0.1 M HCl or 70% HCOOH), 40 °C, 2 days, 60–80%.

Generally, the cleavage of peptides and proteins guided by transition-metal complexes proceeds through stoichiometric reactions. However, in 1996, Kostic and Parac found that *cis*-[Pd(en)(H_2_O)_2_]^2+^ has potential to rupture the H-selective peptide bond of a series of di- and tri-peptides bearing a histidine residue in a catalytic manner.^108^ When a catalytic amount of *cis*-[Pd(en)(H_2_O)_2_]^2+^ was mixed with the peptide solution at pH 1.46, five NMR detectable complexes were formed spontaneously. Together with complexes 79, 80 and 81 (Fig. 6), another two complexes were constructed by the histidinyl peptides by holding two Pd(ii) ions each. When the temperature of the reaction mixture reached 60 °C, the C-terminal amide bond of histidine was fragmented keeping the N-terminal amide bond intact. The rate of the reaction was pH dependent given that the attachment of the catalyst to the peptide was controlled by pH. It was observed that the maximum catalytic efficiency was achieved at pH 1.46. Additionally, the rate constant decreased with an increase in the steric bulk of the amino acid bonded with the carboxylic group of H-residue. Besides, intrapeptide hydrogen bonding through water molecules slowed down the rate of the reaction given that the hydrogen bonded water molecules veiled the catalyst, preventing it from coming closer to the scissile bond. Later, the same research group controlled the regioselective cleavage of H-bearing peptides by the choice of ligands in palladium(ii) complexes. [Pd(H_2_O)_4_]^2+^ cleaves both the N- and C-terminal peptide bonds with respect to the H-residue, where [PdCl_4_]^2−^ cleaves only the C-terminal peptide bond under the same reaction conditions (Fig. 6).^109^ The rate constant of the former reaction was 10 times greater than that of the latter. This reactivity difference is due to steric factors. The [Pd(H_2_O)_4_]^2+^ complex is smaller than [PdCl_4_]^2−^. Consequently, it can come closer to both the scissile bonds and both peptide linkages are fragmented.

**Scheme 13:**
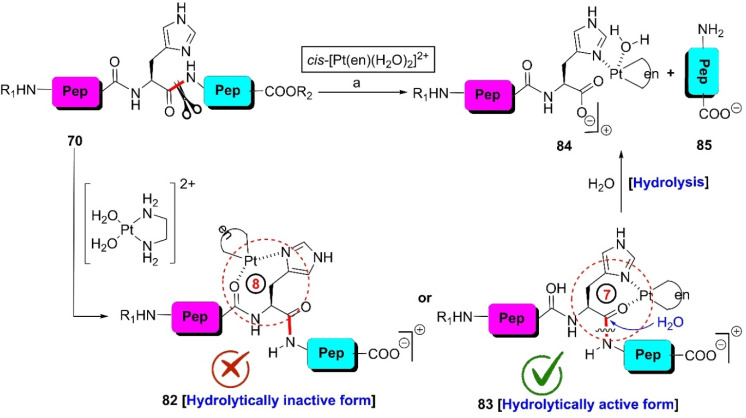
H-selective cleavage of peptide bonds *via* metal complexation. Reaction conditions: (a) peptide :  Pt(ii)-complex = 1 : 1 or 1 : 2, H_2_O, pH 2.65, 60 °C.

In 2010, Hong *et al.* could rapture the C-terminal peptide bond of the H-residue in a series of tripeptides applying a stoichiometric amount of Pt(ii)-complexes (pH 2.65) at 60 °C.^110^ Histidinyl peptide 70 has the probability to construct complex-82 and complex 83 (Scheme 13). Between the two formations, 83 is stereochemically preferable and it is hydrolytically cleavable. As a result, the reaction goes through the formation of complex 83, and upon hydrolysis furnishes products 84 and 85. However, this protocol becomes useless if the tripeptides contain at least one cysteine or methionine residue. Sometimes, an excess amount of costly Pt(ii)-complex is required, which reduces the utility of this protocol.

**Scheme 14:**
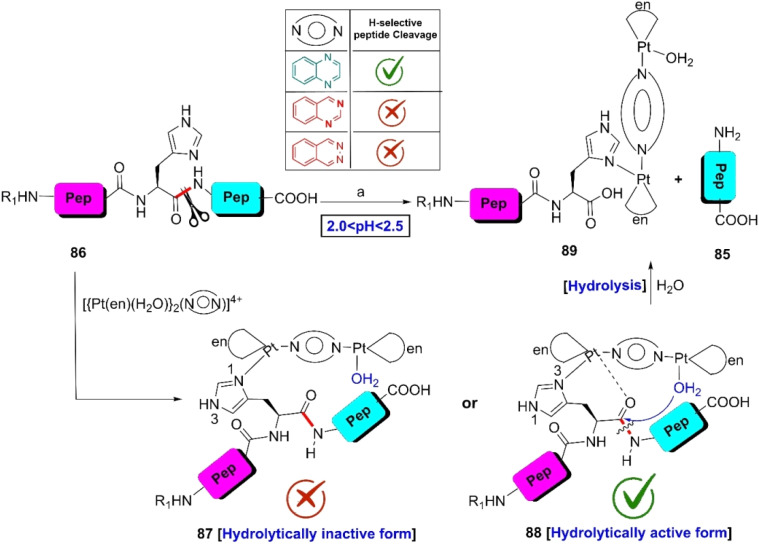
H-selective cleavage of the peptide bond *via* metal complexation: role of ligands. Reaction conditions: (a) D_2_O, pH 2.0 < pH < 2.5, 37 °C.

Recently, Rajkovic *et al.* designed and synthesized three dinuclear platinum(ii) complexes by incorporating bidentate quinoxaline (qx), quinazoline (qz), phthalazine (phtz) ligands and investigated their catalytic behavior towards methioninylglycine and histidinylglycine at a pH of 2.0 < pH < 2.5 at 37 °C.^111^ Similar to platinum(ii) complexes^112,113^ with pyrazine and pyridazine ligands, all three complexes coordinated with the methioninyl side chain of Ac–M–G and split the C-terminal amide bond. However, in the case of Ac–H–G, only the Pt(ii) complex with the quinoxaline-bridging ligand could fragment the C-terminal peptide bond of the histidine residue to yield peptide fragments 85 and 89 (Scheme 14).

Applying [Pd(H_2_O)_4_]^2+^, *cis*-[Pd(en)(H_2_O)_2_]^2+^ and *cis*-[Pd(dtco-OH)(H_2_O)_2_]^2+^ complexes at pH 3.2, Zhu *et al.* could split myoglobin by digesting it for 24 h at 60 °C.^114^ SDS-PAGE electrophoresis and HPLC separation followed by ESIMS and MALDI-TOF experiments of the fragmented protein revealed that 13 selective sites were split, which were clustered around some of the histidine residues. However, most of the fragmented sites were ensured by methionine and arginine residues. Recently, in 2020, Jiao *et al.* improved this protocol by utilizing the binuclear [Pd_2_(μ-O–L–H)(μ-Cl)](ClO_4_)_2_ (L = 2,6-bis(*N*-2′-aminoethylaminomethyl)-*p*-cresol) complex, which cleaves the second upstream peptide bond from the H- and M-residues, avoiding C-orientated hydrolysis, although cysteine also can also serve as an excellent ligand.^115^ Based on theoretical results acquired from B3LYP^70–72^ density functional theory in the Gaussian09 program package using the Lanl2dy and 6-31g(d) basis sets, these investigators confirmed the process of hydrolysis. Synergically, one Pd(ii) center coordinates with the selective site of Mb, and the other one binds with the amide bond and favours nucleophilic attack of the water on the peptide linkage. In the case of cysteine, two Pd(ii)-centers construct a “closed” sulphur-bridge structure, which disfavors the approach of water towards the scissile bond.

#### 3.4.2. Electrochemical method

As is known, histidine can be oxidized at a potential of 1.17 V (*vs.* NHE) at pH 7. The seminal work by the Parsons group exhibited that upon pulse radiolysis of histidinyl dipeptides, electron transfer is possible between the histidine–tyrosine^116^ and histidine–tryptophan^117^ residues, generating a histidinyl radical. Utilizing this radical chemistry, peptides bearing an H-residue may be converted into the corresponding Patchornik intermediate under suitable conditions. However, a report on this is not available in the literature to date.

#### 3.4.3. Chemoenzymatic method

Enzymatic scissoring of histidinyl peptides can be accomplished by applying several proteases depending on the specific sequence present around the H-residue. Two well-known enzymes, trypsin and chymotrypsin, are selectively cleaved at basic residues such as lysine and arginine and AAAs such as F-, W-, and Y-residues, respectively, if they are positioned adjacent to H-residues. However, AAA-selective enzymatic cleavage of amide bonds is out of the scope of this review given that we focused on only chemical, electrochemical and chemoenzymatic methods.

## 4. Future prospect and conclusion

**Fig. 7:**
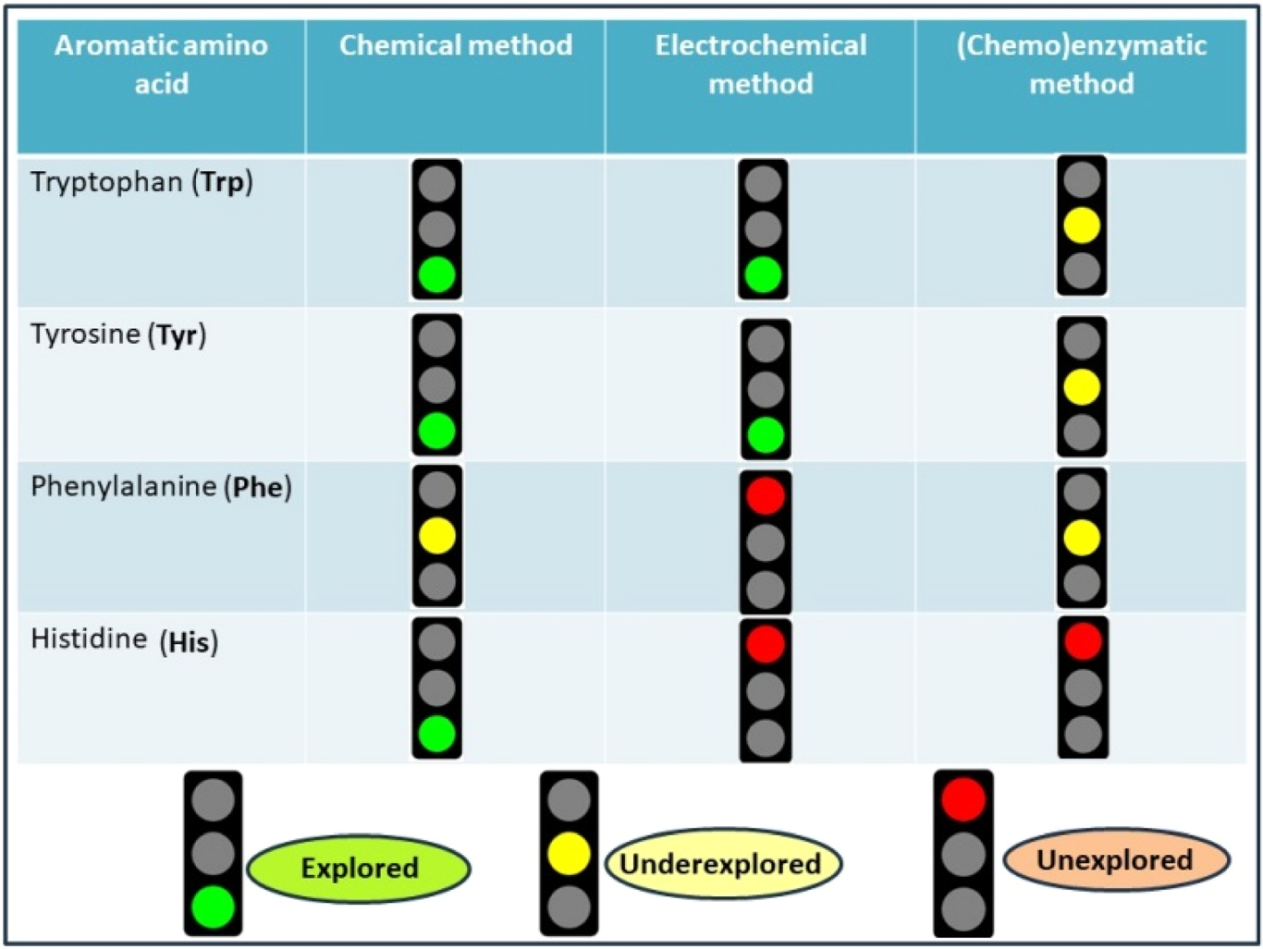
Research achievement *vs.* research gap.

Based on the methodological development of the site-selective cleavage of AAAs in peptides/proteins over the last seven decades (1958–2024), there are tremendous possibilities of further innovation. Fig. 7 depicts the present status of this field in considering the research gaps and achievements achieved over time. According to this figure, it is crystal clear that the electrochemical technique has not been popularized to date for the residue-selective scissoring of peptides, although it has the capability of rupturing peptide bonds *via* clean and green reaction conditions. When a redox-active amino acid is inserted into a peptide or protein, the microenvironment of the amino acid is altered due to the change in the conformation of the protein. This conformational alteration process switches the redox potential of the amino acid side chains. This, by tuning the exact potential of this microenvironment, several selective sites of a protein can be fragmented in a single reaction vessel. Perhaps one day this technique will be beneficial for the instrumental improvement of peptide sequencing machines.

In the last few decades, a fabulous technological improvement has been noticed in the engineering of diverse wild-type enzymes as well as chemoenzymatic processes. However, this has not yet employed in this research territory. Recently, the photochemistry-enabled fragmentation of peptides and proteins has become a catching topic of research. Applying photo-redox reaction, several complex proteins have been split site-selectively. Another review on this topic will be published in due course by our research group.

In most cases, peptide fragmentation reactions are low yielding and the introduction of a sufficient amount of protein in peptide sequencing is not possible. By employing flow-chemistry, this puzzling situation can be solved.

In conclusion, comprehensive methodological improvements in AAA-selective fragmentation have been exhibited over the last 60–70 years. However, there are still huge roadblocks to be overcome. At present, the number of species on Earth is nearly 8.7 million.^118^ Among them, 86% and 91% of species in the Earth and ocean, respectively, are yet to be discovered and analyzed. With their discoveries, chemists and biologists have to encounter new peptides/proteins with complex molecular segments. Analysis of these new proteins will require a huge technological improvement in this research arena. Moreover, the utility of fragmented proteins as drugs and therapeutic agents is increasing. Thus, more sophisticated techniques for the site-selective cleavage of proteins are required at the industrial level. We hope that young researchers will able to tackle all these challenges and this review will inspire the practitioners who are looking to enter this field.

## Data availability

The data that support the findings of this study is already available in the literature.

## Conflicts of interest

There are no conflicts of interest to declare.
